# Nontargeted metabolomics analysis to unravel the anti-biofilm mechanism of Citrocin on *Listeria monocytogenes*

**DOI:** 10.1128/spectrum.01628-24

**Published:** 2025-06-10

**Authors:** Liyao Wang, Wenchao Hou, Hongji Wang, Xuanbo Fan, Hongliang Zhang, Jiaqi Zheng, Liqiong Wang, Yuzhu Han

**Affiliations:** 1 College of Animal Science and Technology, Southwest University 630539 , Chongqing, China; 2 College of Life Science and Technology, Southeast University 12579, Nanjing, China; 3 College of Animal Science and Technology, China Agricultural University 462230 , Beijing, China; 4 Department of Food Science and Nutrition, The Hong Kong Polytechnic University 26680, Hong Kong, China; 5 College of Business, Northwest Normal University 12435, Lanzhou, Gansu, China; 6 Chongqing Key Laboratory of Herbivore Science, Chongqing, China; University of Mississippi, Oxford, Mississippi, USA

**Keywords:** *Listeria monocytogenes*, biofilm, swarming motility, extracellular polysaccharide, metabolomics

## Abstract

*Listeria monocytogenes* biofilm formation is an important cause of cross-contamination in food processing. Citrinin is a potential broad-spectrum antimicrobial peptide. However, the effects of Citrocin on *L. monocytogenes* and its biofilm, as well as the associated mechanisms, remain to be explored. In this study, we evaluated the anti-biofilm effect of the antimicrobial peptide Citrocin on the foodborne pathogen *L. monocytogenes* and analyzed its anti-biofilm mechanism from the perspectives of swarming motility, extracellular polysaccharide production, and metabolite level changes. The results showed that Citrocin had a significant inhibitory effect on the growth of *L. monocytogenes*, with a minimum inhibitory concentration (MIC) of 0.075 mg/mL and a minimum bactericidal concentration (MBC) of 0.15 mg/mL. Citrocin at concentrations of MIC, 2 × MIC, and 4 × MIC could prevent biofilm formation and remove established biofilms. Metabolomics analysis revealed that Citrocin at 0.3 mg/mL caused a significant differential expression of metabolites in biofilms, up- and downregulating 23 and 13 metabolites, consisting mainly of amino acids, organic acids, and fatty acids, respectively. In addition, Citrocin significantly enriched energy and amino acid metabolic pathways, including alanine, glutamate, aspartate metabolism, TCA cycle, and arginine biosynthesis. This work provides potential biofilm regulation strategies and serves as a theoretical basis for the prevention and treatment of listeriosis.

**IMPORTANCE:**

*Listeria monocytogenes* biofilm formation is an important cause of cross-contamination during food processing. We found that Citrocin, an antimicrobial peptide that is widely used in animal feed, has good antimicrobial and anti-biofilm effects against *L. monocytogenes*. We preliminarily explored the anti-biofilm mechanism of Citrocin in terms of swarming motility, extracellular polysaccharide production, and metabolomics. Our work demonstrated that Citrocin is an excellent antimicrobial agent, which is important for the control of food cross-contamination and the preventive treatment of listeriosis.

## INTRODUCTION

*Listeria monocytogenes* is a Gram-positive bacterium that is widespread in nature. It exhibits high tolerance to stressful environments and is commonly found in dairy and meat products, as well as chilled and frozen foods (1, 2). *L. monocytogenes* is a highly hazardous zoonotic foodborne pathogen, with infections manifesting as septicemia, meningitis, and abortion, with a case fatality rate of up to 30% (3). *L. monocytogenes* has the ability to form biofilms on organisms or food processing equipment (4). Biofilms serve primarily as a protective physiological mode for microbial communities, enabling them to persist in the food industry and leading to repeated cross-contamination (5). A number of listeriosis outbreaks have also been associated with biofilm formation (6).

Flagellum-mediated movement is a contributing factor to biofilm formation in *L. monocytogenes* (7). Flagellin A (FlaA), the structural subunit of the flagellum, is encoded by the gene FlaA, and the genes MotA and MotB encode the flagellar motor proteins motA and motB, which regulate flagellar movement (8). Biofilm formation occurs gradually after planktonic bacteria have reached the contact surface (8). For example, planktonic bacteria initially adhere to solid surfaces in a reversible manner through physical interactions such as electrostatic and van der Waals forces (9). Subsequently, the bacterial population increases and facilitates intercellular communication via a variety of signalling pathways, including the quorum sensing (QS) pathway and the two-component regulatory system (10). The two-component regulatory system comprises a sensor, typically a histidine kinase capable of autophosphorylation on conserved histidine residues, and a response regulator to which the phosphate group is transferred (11). This leads to the secretion of various components of extracellular polymeric substances (EPS), promoting the gradual formation of biofilms (12). In general, biofilms are primarily composed of bacteria and extracellular matrix, which comprises extracellular DNA, proteins, and polysaccharides (13). Extracellular polysaccharides are major components of the biofilm matrix and cross-link with eDNA to stabilize the biofilm framework (14). Environmental conditions, bacterial surface characteristics, and external surface properties of materials can all influence the formation of *L. monocytogenes* biofilms (15). Previous studies have revealed that antimicrobial treatments are less effective against biofilms compared to their planktonic counterparts (16), making it particularly challenging to eradicate *L. monocytogenes* once it adheres to various surfaces and forms biofilms (17). The properties of *L. monocytogenes* biofilms have become a major concern in the control of listeriosis originating from food sources (18). Therefore, it is significant to explore the anti-biofilm mechanism of antimicrobial agents used for controlling biofilm.

Citrocin, an antimicrobial lasopeptide containing 19 amino acids, can be heterologously expressed in yeast. Its sequence is similar to that of the microprotein MccJ25, but it is approximately a hundred times more effective than MccJ25. Citrocin contains a right-handed lasso structure with the C terminus passing through a macro loop formed by an isopeptide bond between Gly-1 and Glu-8. The C-terminal tail is spatially locked in place, with Arg-17 positioned above the ring and Tyr-18 below the ring. The right-handed lasso structure confers high stability to Citrocin against chemical, thermal, and protease-induced hydrolysis. Citrocin has demonstrated a significant inhibitory effect on *Escherichia coli* (19). However, no previous research has investigated the anti-biofilm effect of the antimicrobial peptide Citrocin on *L. monocytogenes* or elucidated the mechanism by which Citrocin acts as an anti-biofilm agent.

Therefore, the aim of this study was to assess the antimicrobial properties and anti-biofilm activity of Citrocin against *L. monocytogenes*. To further investigate the anti-biofilm mechanism of Citrocin, we evaluated the microstructure, biofilm metabolic activity, swarming motility, and extracellular polysaccharide production of *L. monocytogenes* treated with different concentrations of the antimicrobial peptide. The effects of both high concentration (MBC) and low concentration (MIC) of Citrocin on *L. monocytogenes* biofilm metabolites were determined by LC-MS-based untargeted metabolomics.

## MATERIALS AND METHODS

### Strains and culture conditions

*L. monocytogenes* L028 was cryopreserved at the Grassland Microbiology Laboratory of Southwest University. Microorganisms stored in glycerol tubes were inoculated onto brain heart infusion (BHI) agar plates and cultured at 37°C for 12 h in an incubator. Single colonies were then picked and inoculated into fresh BHI broth, followed by incubation at 37°C for 12 h in a thermostatic shaker. The antimicrobial peptide Citrocin was provided by Beijing Yinghuier Biotechnology Co., Ltd., with the sequence GGVGKIIEYFIGGGVGRYG and a molecular weight of 1.9 kDa.

### Determination of the antibacterial activity of Citrocin

The inhibitory effect of Citrocin on *L. monocytogenes* was determined using the agar diffusion method. Briefly, 100 µL of microbial suspension (1 × 10^7^ CFU/mL) was evenly spread on a solid medium, and a sterile Oxford cup was placed onto the culture medium coated with bacterial suspension. For each plate, 200 µL of 0.15 mg/mL Citrocin solution was added to the Oxford cup and incubated at 37°C for 16 to 20 h to observe the appearance of inhibition zones. The diameter of the inhibition zones was measured in two perpendicular directions. The same volume of PBS was used as the blank control, and the average diameter of the inhibition zone indicated the antimicrobial effect of the Citrocin against targeted microorganisms (20, 21).

### Thermal stability assay

The thermal stability of Citrocin was investigated. Citrocin solution was treated at 121°C for 20 min. The inhibitory effect against *L. monocytogenes* was then observed, with unheated-treated Citrocin as a negative control (22).

### Minimum inhibitory concentration (MIC) and minimum bactericidal concentration (MBC)

MIC and MBC were determined using the microdouble dilution method (23). Six test tubes were prepared and numbered. Each tube was filled with 2 mL Mueller–Hinton (MH) medium. Dissolved Citrocin was added to test tube 1 and mixed (the final concentration of tube 1 is 0.3 mg/mL), and 2 mL of the mixture was transferred to test tube 2. This procedure was repeated down to test tube 5, and 2 mL of the mixture was discarded. The final concentrations of Citrocin in six tubes were 0, 0.01875, 0.0375, 0.075, 0.15, and 0.3 mg/mL. Next, 100 µL bacterial solution (1 × 10^7^ CFU/mL) was added to all tubes and cultured at 37°C for 24 h. Bacterial growth in the test tubes was evaluated according to the turbidity of the solution. The concentration of Citrocin in the tube at which no macroscopic bacterial growth was observed was defined as the MIC. After cultivating for 18 h, the bacterial solutions from each test tube were inoculated onto MH agar plates, and the peptide concentration at which no visible colony growth was observed was defined as the MBC (24).

### Biofilm inhibition experiment

*L. monocytogenes* was grown to the stationary phase in the BHI broth, and the concentration of the bacterial solution was adjusted to approximately 10^7^ CFU/mL. The bacterial suspension (100 µL) was transferred into a flat-bottom 96-well plate (25). Citrocin solution was added to generate final concentrations of 0.25 × MIC, 0.5 × MIC, MIC, 2 × MIC, and 4 × MIC. As for the negative control, the mixture composed of PBS and the bacterial suspension was added to a set of wells, while wells containing uninoculated BHI broth were used as the blank control. The 96-well plate was incubated at 37°C for 48 h to allow *L. monocytogenes* to form biofilms under static conditions. Planktonic bacteria were removed by rinsing twice with PBS. The samples were air-dried at 37°C for 30 min and stained with 0.1% crystal violet at room temperature for 30 min. The staining solution was then removed, and the wells were washed three times. The biofilm was dissolved with 95% ethanol (200 µL/well) at 37°C for 3 h, and OD_595_ values were measured using a microplate reader (26, 27).

### Biofilm elimination experiment

#### Determination of biofilm biomass

Quantification of biofilms was performed by crystal violet staining. Bacteria grown to the stationary phase were transferred to 96-well plates and cultured at 37°C for 48 h to form mature biofilms. Planktonic bacteria were removed by rinsing twice with PBS. Different concentrations of Citrocin solution were then added and incubated for 4 h, followed by rinsing twice with PBS. Biofilm quantification was carried out as described in the Biofilm Inhibition Experiment section.

#### Biofilm metabolic activity assay

The biofilm metabolic activity was determined using the XTT cell viability assay (28). Following treatment of the biofilms with different concentrations of Citrocin solution, 10 µL XTT and 50 µL PBS were added to each well. The OD_450_ values were measured using a microplate reader after 24-h incubation at 37°C (29).

### Determination of exopolysaccharide content

#### Preparation of the standard curve

For the glucose standard curve, 0.4, 0.6, 0.8, 1.0, 1.2, 1.4, 1.6, and 1.8 mL glucose solution (40 mg/L) were taken, and distilled water was added to reach a final reaction volume of 2.0 mL. Then, 1.0 mL 6% phenol and 5.0 mL concentrated sulfuric acid were added to each tube, mixed, and allowed to stand for 30 min. The absorbance of all tubes was measured at 490 nm using distilled water as the blank in a spectrophotometer (30).

#### Preparation of crude polysaccharide

The bacterial solution cultured overnight was centrifuged to remove the bacteria, and the resulting supernatant was mixed with 95% ethanol in a volume ratio of 1:3. After precipitating overnight at 4°C, the mixture was centrifuged at 24,200*×g* for 10 min to collect the pellet. The absorbance was measured based on the standard curve described in the Biofilm Elimination Experiment section to obtain the polysaccharide concentration (31).

### Swarming motility assay

Citrocin solution was mixed into a semisolid medium to achieve final concentrations of 0.25 × MIC, 0.5 × MIC, MIC, 2 × MIC, and 4 × MIC, respectively. A 5 µL bacterial suspension (10^7^ CFU/mL) was added to the center of the medium and incubated at 37°C for 2 d. The diameter of colony growth in the semisolid medium was observed and measured (32, 33). PBS was considered as the negative control.

### Biofilm structure detection

The biofilm was obtained as described in the Minimum Inhibitory Concentration (MIC) and Minimum Bactericidal Concentration (MBC) section, and crystal violet staining was performed to observe the changes in biofilm structure under different concentrations of Citrocin using a light microscope.

### Changes in metabolite levels

#### Collection of biofilms

*L. monocytogenes* was inoculated into BHI medium at a concentration of 10^7^ CFU/mL. The bacterial solution was added to cell culture plates with a sample volume of 30 mL per well and incubated at 37°C for 48 h. Biofilms were collected using a cell scraper and transferred to 2-mL centrifuge tubes to ensure a wet weight of 100 mg per sample. After collection, biofilms were treated with Citrocin solution at the concentration of 0.075 (MIC) and 0.15 (MBC) mg/mL for 4 h. Supernatants were removed after centrifugation at 4°C, and bacterial pellets were obtained and stored at −20°C. Equal PBS-treated biofilms were used as negative controls, with three replicates per group (34).

#### Metabolite extraction

Each sample was accurately weighed to 50 mg and mixed with 400 µL 80% (V/V) methanol aqueous solution. The bacterial cells were lysed at −20°C, vortexed for 30 s, sonicated at 5°C and 40 KHz for 30 min, and then placed at −20°C for 30 min. After centrifugation at 13,000×*g* for 15 min at 4°C, the supernatant was transferred to a sampler vial for on-board analysis, and a quality control sample consisting of a mixture of all test samples was inserted after every three analytical samples (35).

#### LC-MS/MS analysis

Metabolite extracts were analyzed by liquid chromatography–tandem mass spectrometry (LC-MS/MS) methods using an HPLC instrument (Ultimate 3000, Dionex, Sunnyvale, CA, USA) with a UPLC Hypersil GOLD C18 column (2.1 × 100 mm, particle size 1.9 µm, Thermo Fisher Scientific, USA) and Q-exact Orbitrap (Thermo Fisher Scientific, USA). The flow rate was 0.2 mL/min, the column temperature was 35°C, and the injection volume was 2 µL. The mobile phase system consisted of solvent A (ultrapure water containing 0.1% formic acid), solvent B (methanol containing 0.1% formic acid), solvent C (ultrapure water containing 0.1% NH_3_), and solvent D (methanol containing 0.1% NH_3_). Elution was performed in positive ion mode with a gradient program as follows: 5% A and 95% B (0–10 min), 5% A and 95% B (10–12 min), 5% A and 95% B (12–13 min), and 95% A and 5% B (13–14 min). The negative mode was set with the following gradient program: 95% C and 5% D (0–2.5 min), 95% D and 5% C (2.5–16.5 min), 95% D and 5% C (16.5–19 min), and 95% C and 5% D (19–20 min) (36). MS/MS spectra were acquired in information-dependent acquisition (IDA) mode using Q Exactive Orbitrap under the control of Xcalibur software (Thermo Fisher Scientific, Waltham, MA, USA). The HESI source operating parameters were as follows: sheath gas, 40 arbitrary units; auxiliary gas, 10 arbitrary units; capillary temperature, 320°C; and full-mass scan (m/z 70–1,050) at a resolution of 70,000. The MS/MS scan mode was set to a data-dependent ms2 (dd-ms2) scan at 35,000 resolutions, the high collision dissociation was regulated to 20/40/60 eV in mode, and the spray voltage was adjusted to 3.5 kV (positive mode)/−2.5 kV (negative mode) (37).

### Data analysis

Microsoft Excel 2016 and GraphPad Prism 8.0.2 (GraphPad Software Inc., San Diego, CA, USA) were used for statistical analysis, and the significance test was conducted using an unpaired *t*-test. Raw data were preprocessed using Compound Discoverer 2.1 (Thermo Fisher Scientific, Waltham, MA, USA) to obtain matching and aligned peak data, and then peaks were aligned to the mzCloud and mzVault databases to acquire qualitative and quantitative results. The preprocessed data were imported into SIMCA-P 14.1 (Umetrics, Umea, Sweden) for principal component analysis (PCA) and orthogonal partial least squares discriminant analysis (OPLS-DA). Potential differential metabolites were screened based on variable importance in projection (VIP) ≥1 and *P* < 0.05. Metaboanalyst 5.0 (https://www.metaboanalyst.ca/) was applied to visualize the data. According to the KEGG database (https://www.kegg.jp/), metabolic pathways were constructed. All experiments were repeated three times. The result was considered statistically significant if *P* < 0.05 (*) and regarded as highly significant when *P* < 0.01(**).

## RESULTS

### Determination of the antibacterial activity of Citrocin and thermal stability of Citrocin

**Fig 1:**
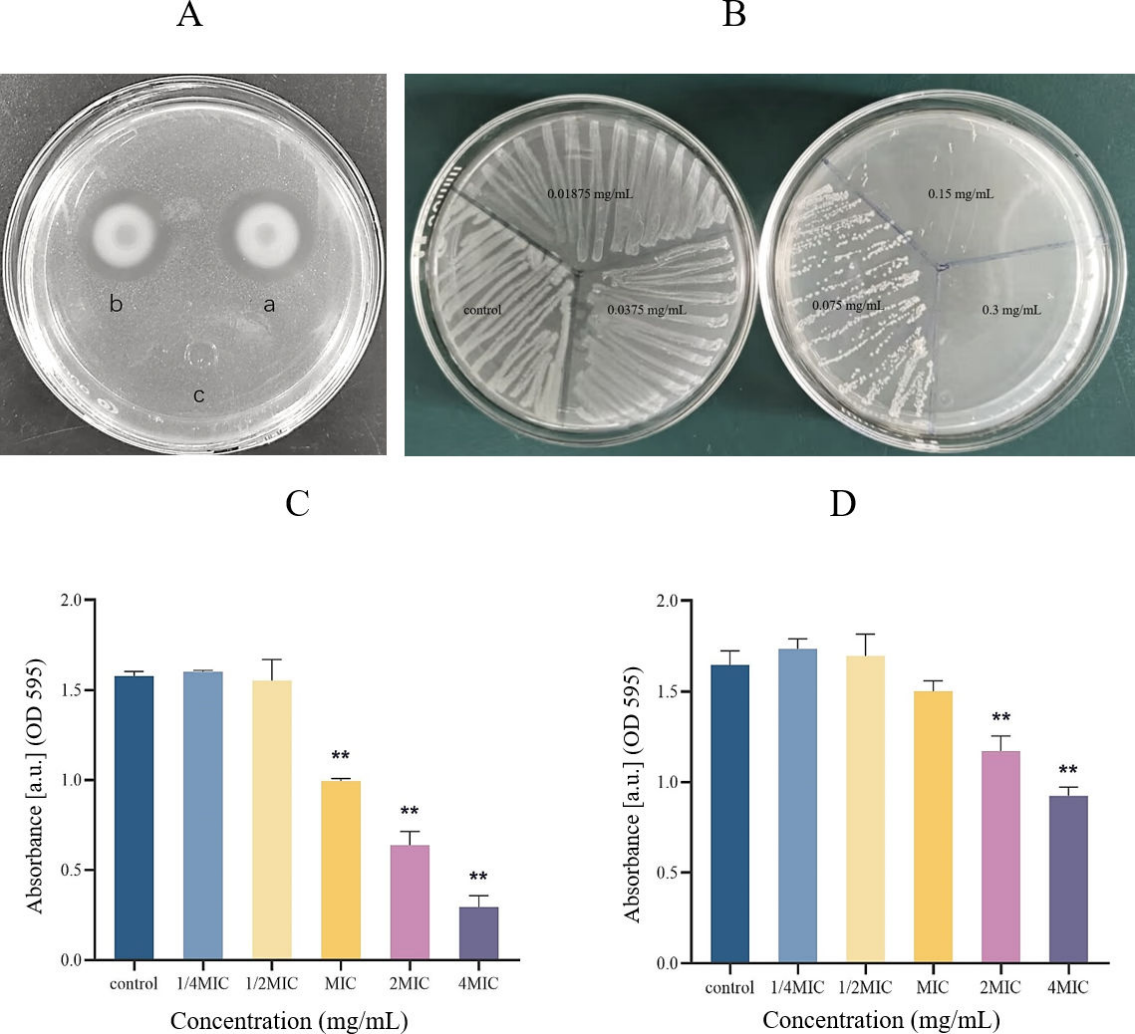
(A) The antibacterial effect of Citrocin against *L. monocytogenes* (a 0.15 mg/mL Citrocin; b 0.15 mg/mL Citrocin after treatment at 121°C; c equal amount of PBS solution). (B) MBC detected by agar plate method. (C) Effect of Citrocin on biofilm formation. (D) Effect of Citrocin on biofilm elimination.

As shown in Fig. 1A, a distinct circle around the Oxford cup supplemented with 0.15 mg/mL of Citrocin demonstrates a strong inhibitory effect on the growth of *L. monocytogenes* compared to the control group. The antibacterial effect of heated Citrocin at 121°C was not significantly different from that of the unheated sample, indicating that the peptide had high thermal stability.

### Minimum inhibitory concentration (MIC) and minimum bactericidal concentration (MBC)

According to the criteria in the Minimum Inhibitory Concentration (MIC) and Minimum Bactericidal Concentration (MBC) section, the MIC was determined to be 0.075 mg/mL. Subsequently, a small amount of bacterial solution was taken from each of the six tubes and inoculated onto BHI agar plates. The number of colonies grown on the plates was fewer than in the control group at a Citrocin concentration of 0.075 mg/mL, and no visible colonies appeared at 0.15 mg/mL (Fig. 1B). Therefore, MBC was recorded as 0.15 mg/mL.

### Biofilm inhibition test

In this study, the effect of Citrocin on *L. monocytogenes* biofilm formation was determined by crystal violet staining (38). Sub-MICs (0.25 × MIC and 0.5 × MIC) of the antimicrobial peptide Citrocin did not inhibit biofilm development; however, higher concentrations (MIC, 2 × MIC, and 4 × MIC) significantly reduced biofilm formation at 37°C (*P* < 0.05) (Fig. 1C).

### Biofilm elimination test

The biomass of *L. monocytogenes* biofilms developed for 48 h at 37°C was measured (39). Sub-MICs (0.25 × MIC and 0.5 × MIC) of Citrocin slightly promoted biofilm formation (Fig. 1D), but the difference was not statistically significant. MIC, 2 × MIC, and 4 × MIC cleared 8.83%, 28.86%, and 43.83% of mature biofilms, respectively, suggesting that a higher concentration of Citrocin or a longer treatment time would be required to completely remove the formed biofilms.

### Determination of biofilm metabolic activity

**Fig 2:**
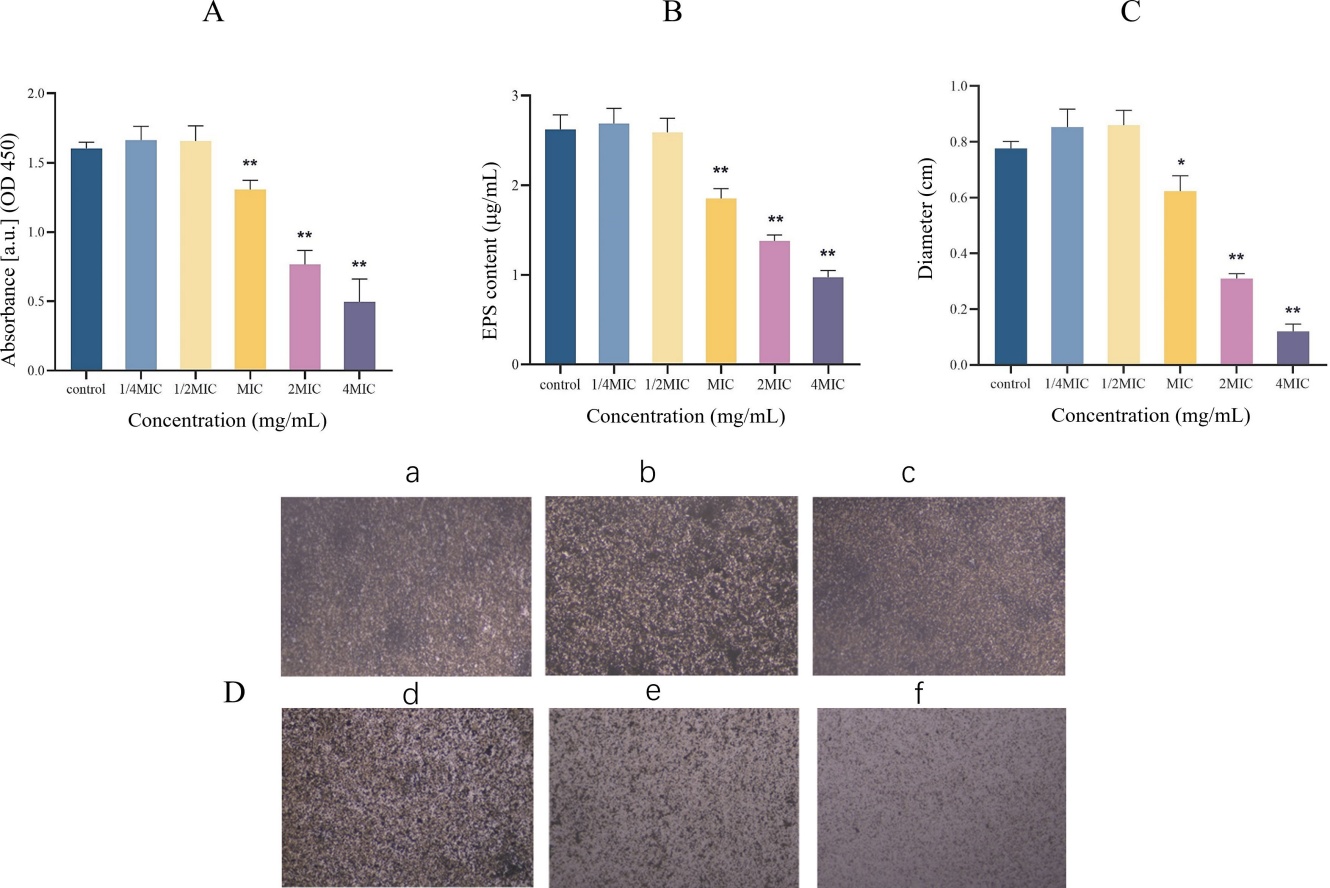
(A) Effect of Citrocin on the metabolic activity of biofilm. (B) Effect of Citrocin on the extracellular polysaccharides of biofilm. (C) Effect of Citrocin on swarming motility of *L. monocytogenes*. (D) The effect of the antimicrobial peptide Citrocin on the structure of *L. monocytogenes*. (*) represents *P* < 0.05; (**) represents *P* < 0.01.

Since crystal violet stains both active and inactive cells (40), we quantified metabolically active cells by XTT analysis (41). MIC, 2 × MIC, and 4 × MIC notably decreased the metabolic activity of biofilm cells (*P* < 0.01) and showed a certain concentration dependence (Fig. 2A). Compared to the Determination of Biofilm Metabolic Activity section, the MIC of Citrocin did not appreciably remove established biofilms (*P* > 0.05) but significantly inhibited the metabolic activity of biofilms (*P* < 0.01).

### Exopolysaccharide content

The equation of the standard curve was determined to be *y* = 0.254*x* − 0.0133, *R*^2^ = 0.9897. *L. monocytogenes* cultured overnight in BHI could produce exopolysaccharides, and the content of exopolysaccharides from untreated bacterial suspension reached more than 2.5 µg/mL (Fig. 2B). Sub-MICs of Citrocin slightly promoted the exopolysaccharide production ability of the bacteria, but the difference was not statistically significant. As the concentration of Citrocin reached MIC, 2 × MIC, and 4 × MIC, exopolysaccharide synthesis in *L. monocytogenes* was significantly inhibited (*P* < 0.01).

### Swarming motility

The swarming motility test showed that *L. monocytogenes* is motile at 37°C. After 48 h of incubation, the colony swarmed to a diameter of 0.78 cm. The motility of *L. monocytogenes* was enhanced by sub-MICs of Citrocin (0.25 × MIC and 0.5 × MIC). The antimicrobial peptide Citrocin at concentrations of MIC, 2 × MIC, and 4 × MIC dramatically decreased the motility of *L. monocytogenes* (*P* < 0.05) in a concentration-dependent manner (Fig. 2C).

### Biofilm structure testing

The biofilm generated by *L. monocytogenes* under different treatments was observed by light microscope. Citrocin caused dense biofilms to become loose and sparse (Fig. 2D).

### Change in metabolite levels

#### Effects of different treatments on the overall metabolic profile of *L. monocytogenes*

**Fig 3:**
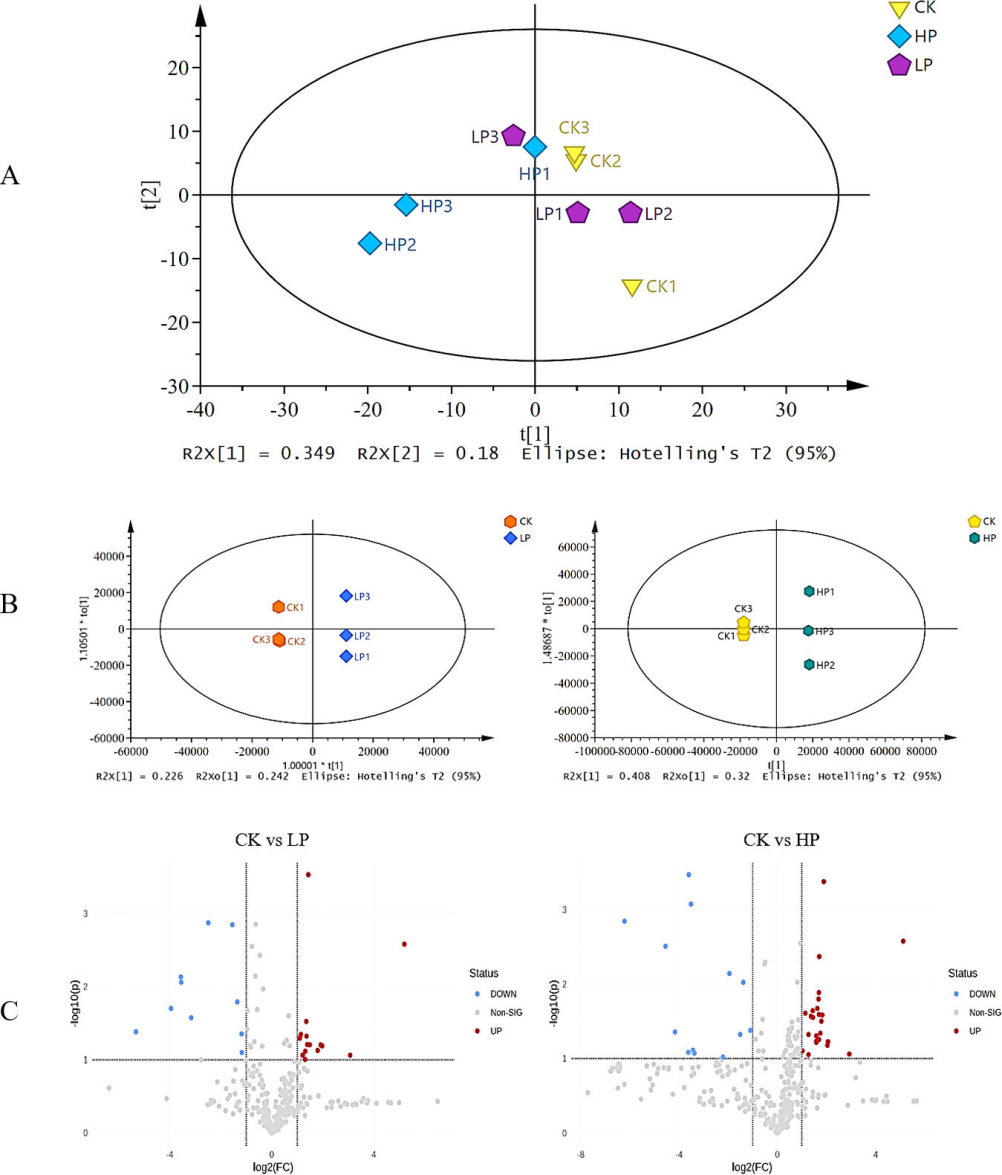
(A) PCA score plots of *L. monocytogenes* biofilm. (B) OPLS-DA score plots in response to different Citrocin treatments. (C) Volcano plots showing the metabolite expression levels between CK, LP, and HP. Blue dots represent downregulated differentially expressed metabolites; red dots represent upregulated differentially expressed metabolites; and gray dots show nondifferentially expressed metabolites. CK, control; LP, 0.15 mg/mL Citrocin; HP, 0.3 mg/mL Citrocin.

Metabolite profiles of *L. monocytogenes* under different treatments were obtained by LC-MS-based metabolomics method, and a total of 350 compounds were detected. The collated data were imported into SIMCA software. In the PCA plot, metabolomics data yielded two principal components (PCs) that explained 52.9% of the total variance (34.9% for PC1 and 18% for PC2). The control (CK) and high peptide (HP) groups were clearly separated (Fig. 3A), whereas the control (CK) and low peptide (LP) groups overlapped greatly, suggesting less variation in their metabolic profiles (42).

OPLS-DA generated pairwise comparisons of the metabolite contributions to maximize separation among groups. As shown in Fig. 3B, samples from different treatments are clearly separated between groups.

The volcano plot (Fig. 3C) revealed that 13 metabolites were significantly upregulated and 10 metabolites were significantly downregulated in CK vs. LP, while 23 metabolites were significantly upregulated and 15 metabolites were significantly downregulated in CK vs. HP. This indicates that high concentrations of Citrocin had a greater impact on the metabolite composition of the bacterial biofilms.

#### Identification of differential metabolites

**Fig 4:**
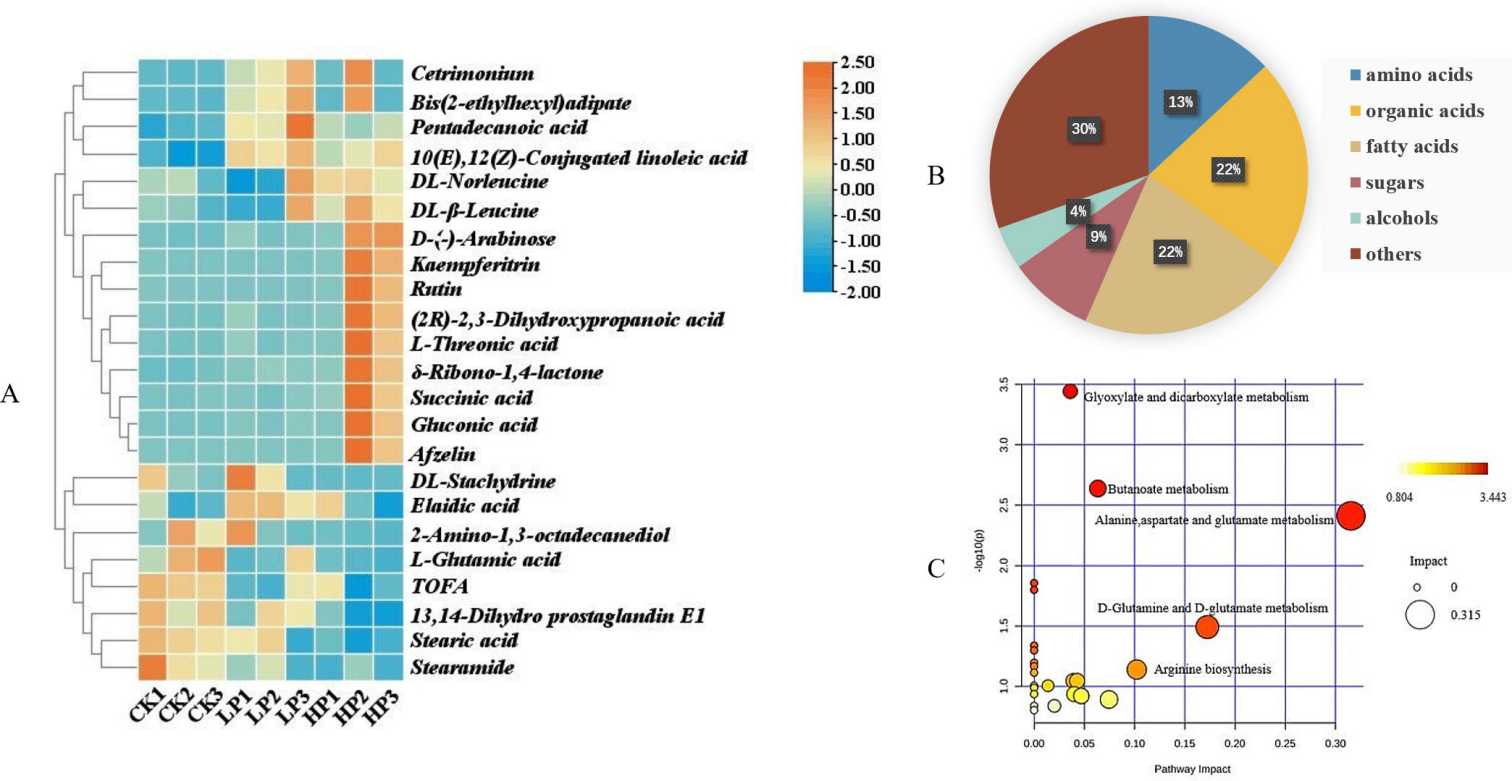
(A) Heat map visualization of differential metabolites in *L. monocytogenes* under different concentrations of Citrocin. The blue and orange represent the low abundance and high abundance ratios, respectively. CK, the control group; LP, 0.15 mg/mL Citrocin; HP, 0.3 mg/mL Citrocin. (B) The classification of metabolites with VIP >1 and *P* < 0.05 in *L. monocytogenes*. (C) Pathway impact analysis between CK and HP. The size of the circle represents the degree of influence of the pathway, with larger circles denoting a stronger influence on the metabolic pathway. The colors of the circles represent −log *P* values for metabolic pathways, with darker colors indicating larger −log *P* values.

VIP (OPLS-DA) >1 and *P* < 0.05 were recognized as the screening criteria. After alignment, seven and 23 differential metabolites were identified in the CK vs. LP and CK vs. HP groups, respectively. Figure 4A presents the heat map of the results for the 23 differential metabolites between the control and Citrocin-treated groups. Among the identified compounds, there were amino acids (13%), organic acids (22%), fatty acids (22%), sugars (9%), and alcohols (4%) (Fig. 4B). Although amino acids and fatty acids belong to the broader category of organic acids, they constitute a significant portion of the metabolites and are thus listed separately. Clearly, the presence of Citrocin significantly altered the content and composition of various metabolites compared to the control, and this effect was dose-dependent.

#### Metabolic pathway analysis

**Fig 5:**
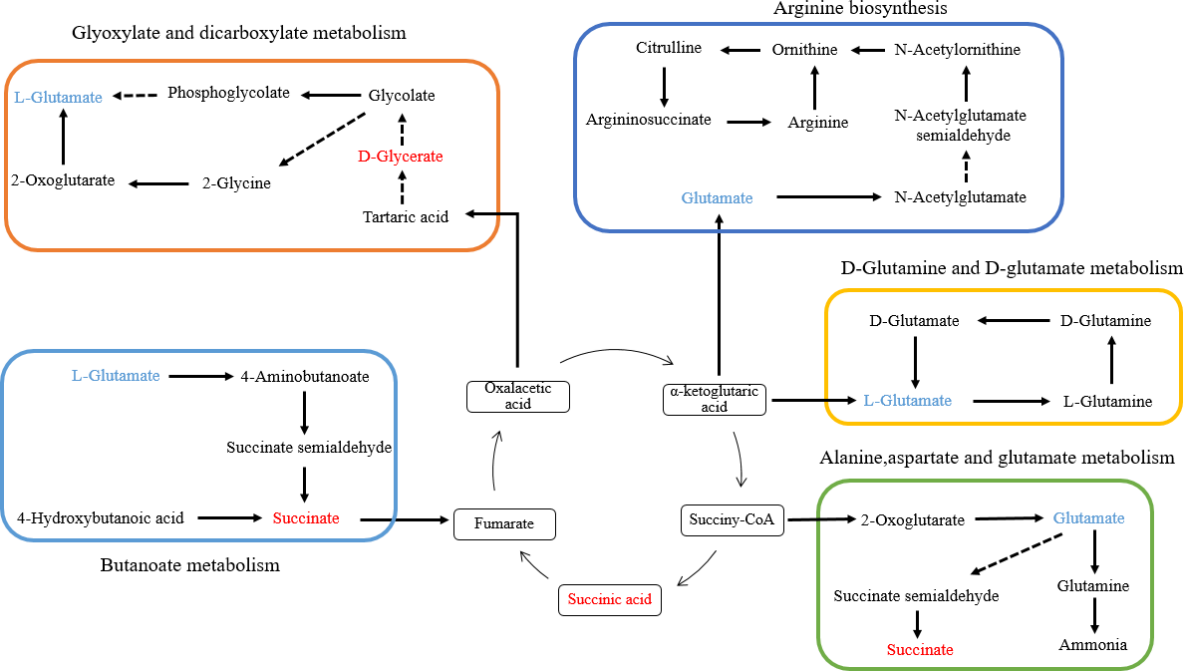
Changes in key metabolites mapped to metabolic pathways between CK vs. HP. The blue color indicates that metabolite content is significantly downregulated, and the red color indicates that the metabolite content is significantly upregulated.

The integrated network of metabolic pathways would provide a more comprehensive understanding of biological processes. The identified metabolites are involved in 24 metabolic pathways. Accordingly, metabolic pathways with relatively significant differences were selected to construct the interconnected network, and the degree of impact is described in Fig. 4C. The metabolic pathway network included alanine, aspartate, and glutamate metabolism; D-glutamine and D-glutamate metabolism; butanoate metabolism; glyoxylate and dicarboxylate metabolism; arginine biosynthesis; and the TCA cycle (Fig. 5). In particular, energy metabolism pathways, such as the TCA cycle and glyoxylate and dicarboxylate metabolism, were upregulated due to the elevation of succinate and glycerate. The downregulation of glutamate abundance led to the inhibition of relevant amino acid metabolism, including alanine, aspartate, and glutamate metabolism; D-glutamine and D-glutamate metabolism; and arginine biosynthesis.

## DISCUSSION

*L. monocytogenes* has been demonstrated not only to be highly invasive but also capable of producing biofilms on both biotic and abiotic surfaces in the food processing industry (43). Microorganisms use biofilms as a means of coping with environmental stress. The formation of microbial biofilms on surfaces in contact with food is considered responsible for approximately 80% of microbial infections (44). Therefore, this study investigated the effect of Citrocin on biofilm formation and removal in *L. monocytogenes*.

Our results demonstrated that sub-MICs (0.25 × MIC and 0.5 × MIC) of Citrocin could not inhibit biofilm formation or remove mature biofilms of *L. monocytogenes*. This is most likely due to the activation of self-defense mechanisms when bacteria respond to external stress (45). Previous studies have indicated that extreme conditions, such as a lack of nutrition, can also stimulate biofilm formation, as cell communities that cooperate for the benefit of the group can develop adaptive mechanisms to overcome extreme conditions (46). Similarly, low concentrations of bacteriostatic drugs allow biofilm cell populations to develop their own effective mechanisms under stress, thereby promoting the growth of biofilms (47). Higher concentrations of Citrocin (MIC, 2 × MIC, and 4 × MIC) were able to inhibit the production of biofilms generated by *L. monocytogenes* and also had a clearance effect on established biofilms. Moreover, higher concentrations of Citrocin were required to remove established biofilm, which is consistent with the findings of Jiang et al. (48) that mature biofilms are obviously more resistant to antimicrobials and are difficult to remove.

In the initial stages of biofilm development, the swarming motility of flagella is essential for bacterial adhesion to contact surfaces. Cell motility is thought to enhance bacterial surface attachment and colonization, contributing to biofilm production (49). Previous studies have shown that single-amplified *L. monocytogenes* mutant strains with ΔflgL and ΔmotA mutations have reduced surface attachment to plant cells and affect the initial attachment phase of the biofilm formation process (50). Additionally, nonmotile strains of *Bacillus subtilis* and *Pseudomonas aeruginosa* generate fragile biofilms (51). The results of this study showed that the motility of *L. monocytogenes* was reduced when treated with Citrocin at concentrations of MIC, 2 × MIC, and 4 × MIC (Fig. 2C). This suggests that Citrocin attenuates the motility of *L. monocytogenes*, affecting the initial colonization of the biofilm, which in turn affects the formation and function of the entire biofilm. Lethality, degradation of flagellum-associated proteins, or other mechanisms are potential ways by which Citrocin affects the swarming motility of *L. monocytogenes*, which we will explore further.

With the attachment of planktonic bacteria, bacterial cells produce EPS to nourish and protect the structural stability of biofilms (52). In the present work, Citrocin was able to drastically reduce the exopolysaccharide production of *L. monocytogenes* (Fig. 2B). Extracellular polysaccharides are the most important constituents of EPS and cross-link with eDNA to stabilize the biofilm framework (53). In addition, EPS production is regulated by biofilm signalling pathways, such as the quorum sensing (QS) pathway and the two-component regulatory system (8). This indicates that Citrocin may disrupt the biofilm signalling pathway to reduce the production of extracellular polysaccharides, thereby collapsing the biofilm framework. However, this hypothesis deserves further confirmation through additional studies.

Metabolomics data indicate that low concentrations of Citrocin did not significantly affect fatty acids and organic acids, but high concentrations induced a distinct downregulation of fatty acids and upregulation of organic acids. Fatty acids are important building blocks of bacterial membranes and are crucial for regulating cellular physiology and function (54). The upregulation and downregulation of fatty acids suggest that the antimicrobial peptide Citrocin clearly perturbed fatty acid metabolism in *L. monocytogenes* biofilm cells. Bacteria actively regulate membrane fluidity by altering lipid composition, which is dominated by fatty acid composition in response to temperature, salinity, pH, and nutrient availability (55). According to the conclusion put forth by Aricha et al. (56), the biophysical characteristics of cell membranes, which are largely controlled by fatty acid composition, can also have an impact on cell adhesion. Thus, bacterial responses to environmental stress are greatly influenced by the composition of cellular fatty acids (54). In this study, metabolomics analysis revealed that *L. monocytogenes* biofilm metabolites after treatment with the antimicrobial peptide Citrocin exhibited substantial changes. Among them, fatty acids accounted for 22% of the total differential metabolites (Fig. 4B). This indicated that Citrocin is capable of regulating the composition of fatty acids in bacterial cells, thereby affecting the fluidity of cell membranes and ultimately altering the way biofilms respond to external environmental threats.

Metabolic changes trigger physiological alterations, and changes in metabolite abundance reflect the inhibition or activation of particular metabolic pathways (57). Upregulation of the abundance of succinate (a TCA cycle intermediate) and D-glycerate (a glyoxylate and dicarboxylate metabolic intermediate) revealed by metabolic pathway analysis indicates upregulation of the TCA cycle and glyoxylate and dicarboxylate metabolism. The TCA cycle and glyoxylate and dicarboxylate metabolism are energy-related pathways, which implies that *L. monocytogenes* biofilms could generate more energy after being exposed to Citrocin. The heat map also showed a relative decrease in the content of fatty acids, demonstrating that the energy produced by the aforementioned two metabolic pathways was not accumulated, but rather consumed to ensure the defensive activity of biofilm communities under stress (58). It is inferred that Citrocin clears biofilms by increasing their energy consumption. In addition, amino acids play an important role in the development of biofilm. For example, glutamate is an essential amino acid for the production of biofilm extracellular matrix (34), and arginine is a basic amino acid that moderates pH decline and maintains a homeostatic environment for biofilm formation (59). Citrocin dramatically downregulated glutamate abundance and inhibited glutamine and glutamate metabolism. Since L-glutamine is the precursor for arginine biosynthesis, reduced glutamate content prevents this process from occurring. Alanine, aspartate, and glutamate metabolism are primarily responsible for nitrogen fixation and the synthesis of protein and even specific enzymes. Therefore, disturbance of this metabolic pathway may lead to impaired function of proteins synthesized by bacteria (60). These data suggest that Citrocin disrupts biofilm-associated amino acid metabolism, thereby inhibiting and eliminating biofilm.

In summary, the antimicrobial peptide Citrocin can inhibit the growth of *L. monocytogenes*, restrain biofilm formation, and eliminate established biofilms. The anti-biofilm mechanism is primarily induced by limiting bacterial cell motility, reducing exopolysaccharide production, accelerating cellular energy metabolism, and suppressing biofilm-related amino acid metabolism. In short, Citrocin is an excellent antimicrobial and anti-biofilm agent, and the investigation of its anti-biofilm mechanism is expected to provide new perspectives for the monitoring and prevention of microbial biofilms in food.

## Data Availability

The authors confirm that the data supporting the findings of this study are available within the article (and/or its supplementary materials).

## SUPPLEMENTAL MATERIAL

Data S1 spectrum.01628-24-s0001.xlsx Raw mass spectrometry data.

ASM does not own the copyrights to Supplemental Material that may be linked to, or accessed through, an article. The authors have granted ASM a non-exclusive, world-wide license to publish the Supplemental Material files. Please contact the corresponding author directly for reuse.
